# Measurement and mitigation of nitrous oxide emissions from a high nitrogen input vegetable system

**DOI:** 10.1038/srep08208

**Published:** 2015-02-03

**Authors:** Shu Kee Lam, Helen Suter, Rohan Davies, Mei Bai, Jianlei Sun, Deli Chen

**Affiliations:** 1 Crop and Soil Science Section, Faculty of Veterinary and Agricultural Sciences, The University of Melbourne, Victoria 3010, Australia; 2 BASF Australia Ltd., Level 12, 28 Freshwater Place, Southbank, Victoria 3006, Australia

## Abstract

The emission and mitigation of nitrous oxide (N_2_O) from high nitrogen (N) vegetable systems is not well understood. Nitrification inhibitors are widely used to decrease N_2_O emissions in many cropping systems. However, most N_2_O flux measurements and inhibitor impacts have been made with small chambers and have not been investigated at a paddock-scale using micrometeorological techniques. We quantified N_2_O fluxes over a four ha celery paddock using open-path Fourier Transform Infrared spectroscopy in conjunction with a backward Lagrangian stochastic model, in addition to using a closed chamber technique. The celery crop was grown on a sandy soil in southern Victoria, Australia. The emission of N_2_O was measured following the application of chicken manure and N fertilizer with and without the application of a nitrification inhibitor 3, 4-dimethyl pyrazole phosphate (DMPP). The two techniques consistently demonstrated that DMPP application reduced N_2_O emission by 37–44%, even though the N_2_O fluxes measured by a micrometeorological technique were more than 10 times higher than the small chamber measurements. The results suggest that nitrification inhibitors have the potential to mitigate N_2_O emission from intensive vegetable production systems, and that the national soil N_2_O emission inventory assessments and modelling predictions may vary with gas measurement techniques.

Globally, agriculture contributes about 58% of total anthropogenic emissions of nitrous oxide (N_2_O), a greenhouse gas 300 times more potent than carbon dioxide^1^. Irrigated vegetable production systems use large nitrogen (N) input which can be susceptible to substantial N loss, including N_2_O emission^2^. The recovery of the applied N by vegetable crops rarely exceeds 50% and can be as low as 20%^3^. Nitrification inhibitors inhibit nitrification and subsequent denitrification, thereby reducing N_2_O production^4^. A global meta-analysis suggests that nitrification inhibitors reduce N_2_O emissions by 31–44% in agricultural systems^5^. Nevertheless, there is a dearth of information on the effect of nitrification inhibitors on N_2_O emission from intensive vegetable production systems, and large-scale measurements with the use of a micrometeorological technique have not been conducted. This information is needed for efficient N management and mitigation of agricultural greenhouse gas emission. We therefore conducted a field experiment to investigate the effect of a nitrification inhibitor 3, 4-dimethylpyrazole phosphate (DMPP) on N_2_O emission from a vegetable farm in Boneo (38.4°S, 144.9°E) Victoria, Australia. In addition to the widely used closed chamber method, we quantified paddock-scale N_2_O fluxes with and without DMPP application using an open-path Fourier Transform Infrared spectroscopy (FTIR) in conjunction with a backward Lagrangian stochastic (bLS) model^6^.

## Results and Discussion

**Figure 1:**
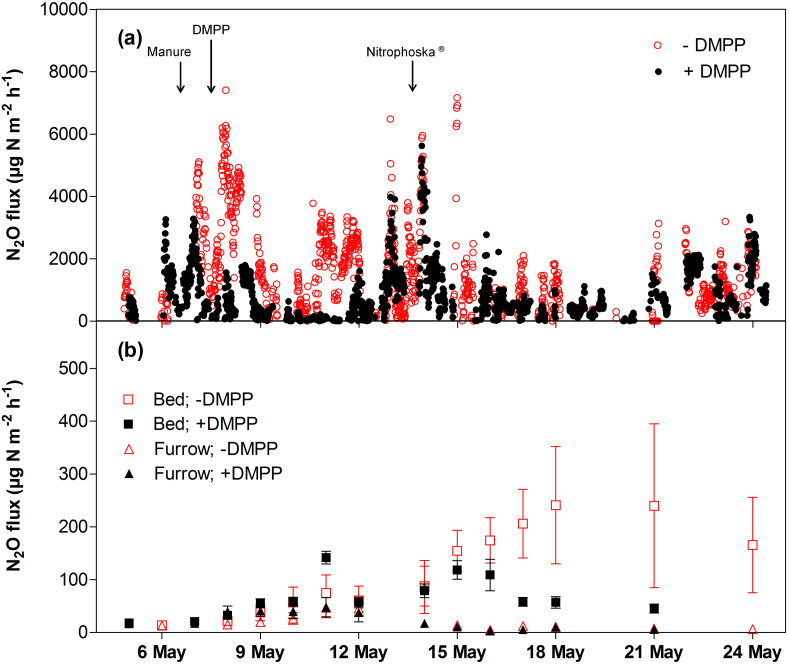
Effect of DMPP application on N_2_O emission measured by (a) open-path FTIR spectroscopy (simulated at background N_2_O concentration enhancement of 5 nmol mol^−1^ with the bLS model) and (b) closed chamber method from the bed and the furrow. Values are the means of five replicates for each treatment. Vertical bars indicate standard errors.

Nitrous oxide emission from the celery paddocks increased after the application of chicken manure and Nitrophoska® regardless of DMPP treatment (Fig. 1). The emission was mostly from the celery growing bed where N was applied rather than from the furrow (Fig. 1b). The meteorological and chamber techniques showed that the application of DMPP reduced the N_2_O emission by 37–44% (Table 1). The percentage decrease in N_2_O emission in our study was comparable to that reported in ref. 7, which showed a 40–45% reduction of N_2_O emission (from closed chambers) in a DMPP-treated lettuce-cauliflower farm in Germany. In contrast, a 75% decrease in the emission (from automatic chambers) was noted when DMPP was applied to a broccoli farm in subtropical Australia^8^. The actual N_2_O emission from this broccoli farm was lower than that observed in our study and in ref. 7. Our study and that of ref. 7 were conducted in temperate regions while that of ref. 8 in a subtropical region. The difference in the effectiveness of DMPP in lowering N_2_O production between these studies could be attributed to the actual N_2_O emission and environmental factors such as soil temperature and moisture content, which may affect soil microbial metabolism and/or populations^9^.

**Figure 2:**
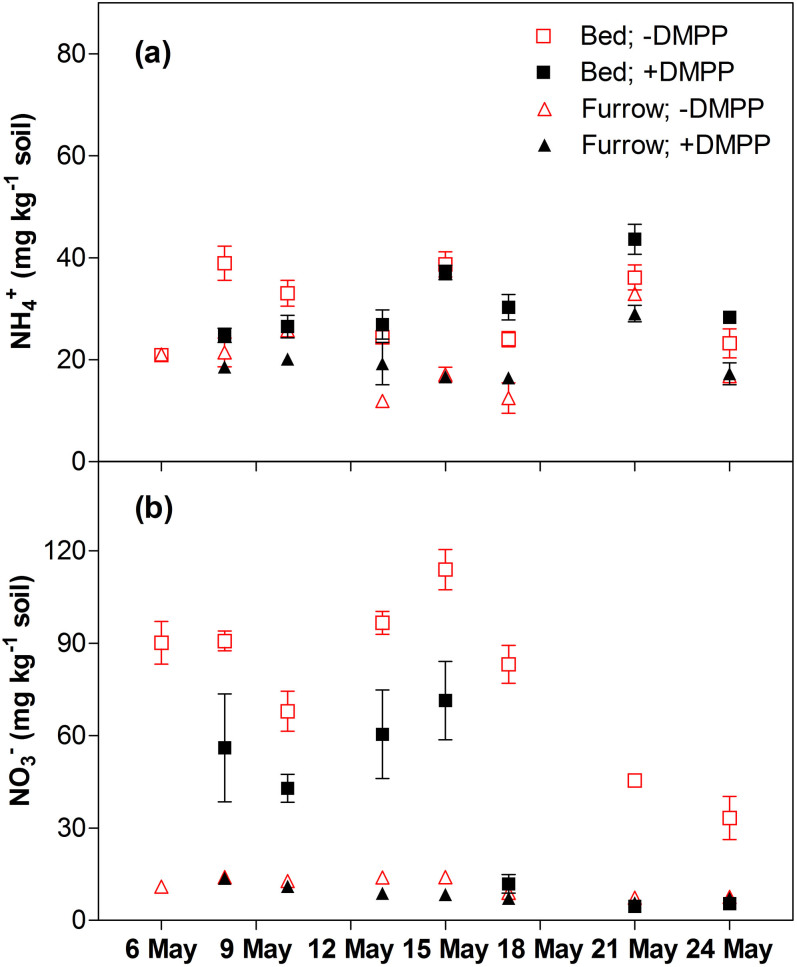
Soil (0–15 cm) (a) NH_4_^+^ and (b) NO_3_^−^ contents from the bed and the furrow with and without DMPP application. Values are the means of four replicates for each treatment.

A nitrification inhibitor lowers N_2_O emission by preventing or slowing the microbial conversion of ammonium (NH_4_^+^) to nitrate (NO_3_^−^) (ref. 4, 10). In our study, soil NO_3_^−^ content in the DMPP-treated celery growing bed was decreased by an average of 49% (*p* < 0.001) (Fig. 2b), which explains why N_2_O emission was lower under DMPP application. The decrease in soil NO_3_^−^ content also suggests that NO_3_^−^ leaching was likely reduced in the paddock treated with DMPP. Soil NH_4_^+^ content did not differ significantly between the control and DMPP treatment (Fig. 2a). Nonetheless, any DMPP-induced difference in NH_4_^+^ content in our study would be small when compared to the substantial NH_3_ volatilisation^11^ resulting from the high rate of surface NH_4_^+^-N application and alkaline soil pH^12^,^13^.

The relative effects of DMPP on N_2_O fluxes were consistent between the two techniques used in our study, despite the absolute flux values measured by these techniques differing by 7- to 40-fold under different background N_2_O enhancement concentrations (Table 1). This difference contrasts with other studies which reported similar magnitude of the fluxes measured by chamber and micrometeorological methods^14^,^15^. Rochette and Eriksen-Hamel^16^ evaluated a data set of 356 studies of chamber measurement of soil N_2_O, and concluded that the flux data might be valid for comparisons between treatments but could be biased estimates of actual fluxes. These findings indicate that the actual N_2_O flux estimates obtained by different techniques are not always in good agreement. The following four explanations for the discrepancy in the actual fluxes we measured between the micrometeorological and chamber techniques are feasible. First, the issue of high spatial variability of N_2_O emission^17^,^18^ was more likely overcome by paddock-scale measurement using a micrometeorological technique which covered all N_2_O emission ‘hot spots'. Second, the temporal or diurnal variability of N_2_O flux was captured by continuous measurements using open-path FTIR spectroscopy while the 1-hour ‘snapshot' measurements by closed chambers might have excluded any sporadic emission peaks^8^,^17^. Third, significant events of N_2_O emission associated with water input might have missed out from chamber measurements particularly for vegetable production systems with substantial irrigation. Fourth, the micrometeorological data filtering process for the bLS model excluded data associated with low wind speed (friction velocity ≤0.15 m s^−1^)^19^ which was more common at night when the flux was low, thereby possibly overestimating the daily N_2_O fluxes. The lack of simultaneous measurements of background N_2_O concentration in our study resulted in variation in the absolute N_2_O fluxes simulated by the bLS model, but this variation did not invalidate the treatment effects, which was the focus of our study.

In summary, our results indicate that N_2_O emission from an intensive vegetable farm can be mitigated by using a nitrification inhibitor. The N_2_O fluxes measured by different techniques should be interpreted carefully when making assessments on an absolute scale in national inventories of soil N_2_O and in model estimates from agricultural systems. Further study is required to substantiate the contrasting difference in gas measurements by these techniques under a range of agricultural systems and climatic conditions.

## Methods

Celery crops were transplanted on 6 and 7 April 2013 at the 4–5-leaf stage and received post-transplant fertilizer (calcium nitrate) at 39 kg N ha^−1^. The gas measurement was conducted between 6 and 24 May. This was the most intensive period of N application, which encompassed surface application to the celery growing beds of chicken manure (3.4% N) at 255 kg N ha^−1^ on 7 May and Nitrophoska® (12% N) at 39 kg N ha^−1^ on 14 May. The average minimum and maximum temperatures during the study period were 7.7°C and 17.4°C, respectively, with a total rainfall of 108 mm^20^. Two paddocks (243 m × 192 m) in the farm were used for this study, one for the control and the other for DMPP treatment (applied at 6.64 kg ha^−1^ on 8 May). The soil is classified as a Tenosol^21^ with 91% sand. The soil (0–15 cm) has a pH (1:5 soil: water) of 7.9 and contains 0.64% organic carbon. The NH_4_^+^ or NO_3_^−^ content did not differ between the two paddocks five days after the celery transplant, and ranged from 15.7–16.1 mg N kg^−1^ and 11.4–11.7 mg N kg^−1^, respectively.

Details of the technique of open-path FTIR spectroscopy in conjunction with the WindTrax model have been described in ref. 11. Briefly, an open-path FTIR spectroscopic system (Matrix-M IRcube, Bruker Optik GmbH) was established at the centre of each paddock at 1.2 m height with a path length of 98 m. Nitrous oxide concentrations were continuously measured at 3-min intervals. Measured spectra were analyzed at spectral region of 2300 cm^−1^ using a Multi-Atmospheric Layer Transmission model^22^ and the high-resolution transmission molecular absorption database^23^. A three-dimensional sonic anemometer (CSAT3, Campbell Scientific) was located at the centre of each paddock at 2.3 m height. Ten-minute averaged micrometeorological data, including wind components covariance and variations, wind speed, wind direction and air temperature, were recorded at 10 Hz. The fluxes of N_2_O were simulated at 10-min intervals using the bLS model (WindTrax 2.0, Thunder Beach Scientific) based on any enhancement in N_2_O concentration measured in the paddock compared to that outside the paddock (background concentration). While no simultaneous measurements of background N_2_O concentration were conducted throughout the study period, the flux calculations would have been affected by any diurnal variation in background concentration. Therefore, based on an average diurnal variation in background N_2_O concentration (10 nmol mol^−1^; observed across one week prior to manure application), we estimated the N_2_O fluxes using three background concentrations (0, 5 and 10 nmol mol^−1^ enhancement).

The fluxes of N_2_O were also measured using closed chambers^24^ (25 cm diameter, 15 cm height) at the control and DMPP-treated paddocks, both in the bed and furrow areas, with five replicates randomly located at where the open-path FTIR measurements were taken. The chambers were inserted to a soil depth of 5 cm. On each sampling day, gas samples (20 mL) were collected between 1300–1600 h at 0, 30 and 60 minutes after chamber closure using a gas-tight syringe, transferred into evacuated 12 mL vials (Exetainer®, Labco Ltd.) and analysed by gas chromatography (Agilent 7890A). The flux rates of N_2_O were calculated as described in ref. 25. Soil (0–15 cm) samples were collected across each paddock from the bed and furrow areas using a 2.5 cm internal diameter corer. Four replicate samples (a composite of 15 soil cores) were collected by traversing each quadrat of the paddock from the corner to the centre. Subsamples (20 g, dried at 40°C, <2 mm) were extracted with 100 mL 2 M potassium chloride^26^. The concentrations of NH_4_^+^ and NO_3_^−^ in the filtered extract were determined colorimetrically by a segmented flow analyser (Skalar SAN^++^). Data of gas fluxes obtained from closed chambers and mineral N were analysed with MINITAB 16 statistical package using a General Linear Model analysis of variance.

## Author Contributions

D.C., H.S., R.D. and S.K.L. designed the investigation. S.K.L., H.S., M.B. and J.S. conducted the field experiment. S.K.L., H.S., D.C. and M.B. interpreted the data. All authors were involved in writing the paper.

## Figures and Tables

**Table 1:** The effect of DMPP application on average N_2_O flux measured by open-path FTIR spectroscopy (with 0, 5 and 10 nmol mol^−1^ background enhancement) and closed chamber techniques across 8–24 May

	N_2_O flux (g N ha^−1^ d^−1^)			
	Open-path FTIR (with background enhancement, nmol mol^−1^)			Chamber^a^ (mean ± SE, *n* = 5)
	0	5	10	
Control	1053.0	317.8	168.2	23.7 ± 6.8
DMPP	589.5	197.3	106.9	14.7 ± 2.3
% change due to DMPP application	−44	−38	−37	−38 (*p* = 0.2)

^a^calculated based on the ratio of the width of the bed to that of the furrow (3:1).
